# Comments on ‘Bisphosphonate and statin: adverse effects of co-medication on wound healing in *in vitro models* of periodontal tissues’

**DOI:** 10.2340/aos.v85.46238

**Published:** 2026-06-10

**Authors:** David Szaraz, Vojtech Perina, Petra Borilova Linhartova

**Affiliations:** a Clinic of Maxillofacial Surgery, University Hospital Brno, Brno, Czechia; b Faculty of Medicine, Masaryk University, Brno, Czechia; c RECETOX, Faculty of Science, Masaryk University, Brno, Czechia

**Keywords:** Bisphosphonate, alendronate, statin, simvastatin, osteonecrosis

## Abstract

This is a comment on the article ‘Bisphosphonate and statin: adverse effects of co-medication on wound healing in *in vitro* models of periodontal tissues’ https://doi.org/10.2340/aos.v85.45585

Dear Editor,

The recently published article titled ‘Bisphosphonate and statin: adverse effects of co-medication on wound healing in *in vitro* models of periodontal tissues’ from Småland-Reksten et al. [1] sparked great interest in our research team. Their research focused on the much-needed topic of the impact of concomitant influence of simvastatin (SIM) and alendronate (ALN) on wound healing. The study revealed that not only do these two drugs seem to have a synergistic adverse effect on bone turnover, but also SIM alone can in higher doses alter wound healing just as much as the combination of the two drugs.

Two years ago, we published a case report on a rare case of medication-related osteonecrosis of the jaw (MRONJ), which occurred just after a single low dose of denosumab (DMB) [2]. In that paper, we argued that previous atorvastatin and ALN medication predisposed the patient, yet DMB was the trigger of MRONJ development. Based on a review of four papers of MRONJ after statin medication in our article [3–6], we also proposed that statins probably have a pleiotropic effect in a dose-dependent manner. Based on that observation, we speculated that lower doses of statins and short-term medication support angiogenesis, while higher doses and long-term medication inhibit it. In a recent *in vivo* study using a mouse model, Bae et al. observed that an antibody against vascular endothelial growth factor (VEGF, one of the key factors in angiogenesis) delays the healing of osteomucosal wounds but does not cause bone necrosis on its own [7]. Rather, it is the combination of osteoclast suppression and angiogenesis inhibition that is considered a risk factor for the development of MRONJ [7].

It is interesting to see in the study by Småland-Reksten et al. that VEGF peaked after the first day of SIM exposure (whether alone or combined with ALN). This may explain why some studies reported beneficial effect of statins on healing where statins were usually given in a single dose [8–10]. However, as this paper shows, the continuation of SIM actually causes a decrease in VEGF. We are happy to see that we found support to our hypothesis [2] in this recent paper, suggesting that duration might be just as important as dosage. In fact, Adachi et al. observed that experimental models of MRONJ in which higher doses of fluvastatin (but only in a single dose) were administered had a better overall outcome compared to those with lower doses [9]. However, besides the effect of statins on VEGF, it is noteworthy that statins and ALN both act through the mevalonate pathway [5], constituting a crucial signaling pathway in bone turnover. This may further explain the synergistic effect of statins and bisphosphonates.

It would be also interesting to investigate the possible interactions between SIM and DMB or SIM, bisphosphonates, and DMB on both soft and hard tissues in oral cavity. We would like to congratulate the research team for the great success achieved in their study. We hope to see more research published on this topic.
